# Delayed presentation and diagnosis of breast cancer in African women: a systematic review

**DOI:** 10.1016/j.annepidem.2017.09.007

**Published:** 2017-10

**Authors:** Carolina Espina, Fiona McKenzie, Isabel dos-Santos-Silva

**Affiliations:** a International Agency for Research on Cancer (IARC), Lyon, France; b Department of Non-Communicable Disease Epidemiology, London School of Hygiene and Tropical Medicine, London, UK

**Keywords:** Breast cancer, Africa, Delayed presentation, Delayed diagnosis, Late-stage breast cancer

## Abstract

**Purpose:**

Africa has low breast cancer incidence rates but high mortality rates from this disease due to poor survival. Delays in presentation and diagnosis are major determinants of breast cancer survival, but these have not been comprehensively investigated in Africa.

**Methods:**

MEDLINE, Embase, and Global Health were searched to identify studies reporting on delays in presentation and/or diagnosis of breast cancer published between January 1, 2000 and May 31, 2016. Data were synthesized in narrative, tabular, and graphical forms. Meta-analyses were not possible due to between-study differences in the way delays were reported.

**Results:**

Twenty-one studies were included in the review. Study-specific average times between symptom recognition and presentation to a health care provider ranged from less than 1 to 4 months in North Africa and from less than 3 to greater than 6 months in sub-Saharan Africa. Study-specific average times from presentation to diagnosis were less than 1 month in North Africa but ranged from less than 3 to greater than 6 months in sub-Saharan Africa. Reported reasons for these delays included patient-mediated (e.g., socioeconomic factors) and health system–mediated factors (e.g., referral pathways).

**Conclusions:**

This systematic review revealed marked delays in presentation and diagnosis of breast cancer in Africa. Identification of their drivers is crucial to the development of appropriate control strategies in the continent.

## Introduction

Women in Africa currently have one of the lowest incidence rates of breast cancer worldwide [1]. However, the burden from this cancer is expected to increase markedly in the next decades. A growing aging population alone, that is, assuming incidence rates will remain constant, will lead to estimated 119,918 new cases in 2030, a near doubling in the number of incident cases over 20 years [2]. The increase will be even more marked as incidence rates are likely to rise due to the adoption by African women of more westernized lifestyle profiles, particularly reproductive patterns characterized by late age at first full-term pregnancy, lower parity, reduced lifetime, breastfeeding duration, and increases in postmenopausal weight [3].

Despite breast cancer incidence rates being still relatively low in Africa, mortality rates from this disease are as high, or higher, than in high-incidence countries due to poor survival [1]. Furthermore, the proportion of breast cancer cases and deaths at premenopausal ages is higher in Africa than in high-income countries (HICs), where disease incidence is highest, reflecting the younger age structure of the continent's population and possibly also distinctive risk factors and/or tumor characteristics. Consequently, breast cancer in Africa disproportionately affects women in the prime of their lives, and hence, it has particularly marked familial, societal, and economic consequences.

A recent systematic review [4] shows that a high proportion of breast cancer patients in sub-Saharan Africa (SSA) are diagnosed with late-stage disease leading to poor survival [5]. Studies from HICs have shown that delays between onset of symptoms and start of treatment are main determinants of late-stage presentation and poor survival [6]. Previous studies have attempted to examine delays in breast cancer presentation, diagnosis, and treatment in Africa [5], [7], but, to our knowledge, these have not been comprehensively investigated across the continent. Knowledge of the length of time intervals between symptom recognition, presentation, diagnosis, and start of treatment—and of the factors that may influence them—is the key to the development of strategies to shorten them. Therefore, we conducted a systematic review to investigate delays in presentation and diagnosis of breast cancer in Africa and their determinants.

## Materials and methods

### Conceptual framework

**Fig. 1:**
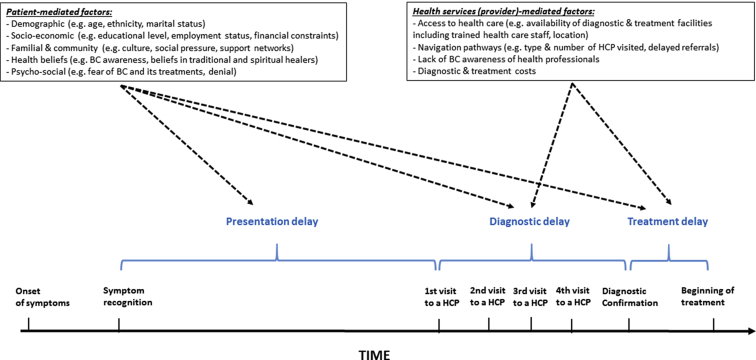
Presentation, diagnostic, and treatment delays in breast cancer. BC = breast cancer; HCP = health care provider.

Figure 1 depicts a patient's trajectory from the moment she first notices symptom(s) to the time when treatment starts and the factors that may affect her journey. In HICs with free universal access to health care, the delay from a woman first noticing potential symptoms of breast cancer to her presentation to a health care provider is labeled as “patient delay” as it is essentially driven by patient-mediated factors. In contrast, the time from first medical consultation to the beginning of definitive treatment is labeled as “provider delay” as it is driven predominantly by health system–mediated factors. However, in many African settings, the picture is likely to be far more complex as delays in both presentation and diagnosis are likely to result from a complex interplay between patient-mediated and health system–mediated factors. For instance, a woman may delay presentation not only because of her lack of breast cancer awareness but also because of the unavailability of health care providers in her area of residence. Similarly, a woman who first presents with a suspicious cancer may delay diagnosis due to fear of its consequences (e.g., mastectomy, death). In this review, we will consider presentation delays as the time interval from symptom recognition to presentation to the first health care provider, diagnostic delays as the time interval between presentation and breast cancer diagnosis, and treatment delays as the time interval between diagnosis and start of cancer treatment. These terms do not carry any judgment on whether these delays are primarily induced by patient-mediated or provider-mediated factors.

### Search methodology

The Preferred Reporting Items for Systematic Reviews and Meta-Analyses statement guidelines [8] were followed to select relevant publications on delays in breast cancer presentation and diagnosis in Africa. Articles were eligible for inclusion in the systematic review if they reported findings from primary research studies conducted in Africa; reported on delays in presentation and/or diagnosis of female breast cancer patients; and were published between January 1, 2000 and May 31, 2016. No language restrictions were imposed. Relevant publications were searched in the electronic databases MEDLINE, Embase, and Global Health. A search strategy using synonyms (including truncations) and subject headings of the search concepts “breast cancer,” “late diagnosis,” “Africa,” and “determinants,” and the Boolean operators “AND” and “OR” was used (Appendix A). All titles and abstracts were screened to identify potentially eligible articles and the full text for these retrieved and critically reviewed articles to assess eligibility and, if eligible, to extract relevant data.

### Data extraction

The data extraction from each eligible article was carried out independently by two reviewers (C.E. and I.d.-S.-S.) using a specifically developed standardized data extraction form. The following information was extracted: the type of catchment population (e.g., country, urban, rural, or mixed); the study design (quantitative, qualitative, mixed); the type of recruitment source (primary, secondary, or tertiary hospital/clinic) and approach (eligibility criteria, recruitment period, type of sample: consecutive or convenience, i.e., opportunistic, sample size); patient (e.g., age) and tumor characteristics (e.g., stage, size, histology, symptoms); source (e.g., patient, medical records) and timing of collection (e.g., before or after diagnosis) of data on delays and their reasons; reported times between symptom recognition, presentation, diagnosis, and start of treatment; and patient-mediated and health system–mediated factors that might have influenced them. Disagreements between the two reviewers were discussed, and a consensus was reached.

### Quality assessment of the eligible articles

The quality of the articles included in the review was assessed independently by the same two reviewers. A standardized quality assessment form was developed, which included parameters to assess the potential for selection and information bias as well as the appropriateness of the analytical methods used, including those for dealing with potential confounders (Appendix B). The overall quality score of an article was expressed as the sum of its parameter-specific scores, which could range from 0 (lowest) to 30 (highest). The higher the score, the higher the methodological quality of the article; the lower the score, the more likely its findings might have been affected by biases.

### Data synthesis

Data were synthesized in narrative, tabular, and graphical forms. Study-specific mean (SD) or median (range), presentation, diagnosis, and treatment delays are presented; if only categorical data were reported in the original publication, we used them to estimate the median, or a weighted mean, whenever possible. Studies differ greatly in the way they obtained information on potential reasons for delays and in the way such data were presented (Appendix C). Most studies simply presented data in a descriptive way (e.g., percentages), but a few used logistic regression methods to estimate crude and/or adjusted odds ratios for delayed presentation, diagnosis, or treatment for each variable examined, with studies using different cutoff points to define such delays (e.g., from ≥2.2 to >6 months for delay in presentation and from >2 weeks to ≥6 months for delays in diagnosis; Appendix C). One study in North Africa [9] reported on delays but only examined factors associated with late stage (III/IV) versus early stage at diagnosis; late stage was taken here as a proxy for delays between symptom recognition and diagnosis. Findings are shown separately for studies conducted in North Africa (i.e., in Algeria, Egypt, Libya, Morocco, Sudan, Tunisia, and Western Sahara) and SSA (i.e., countries in East, Middle, South and West Africa) as defined by the United Nations [10].

## Results

**Fig. 2:**
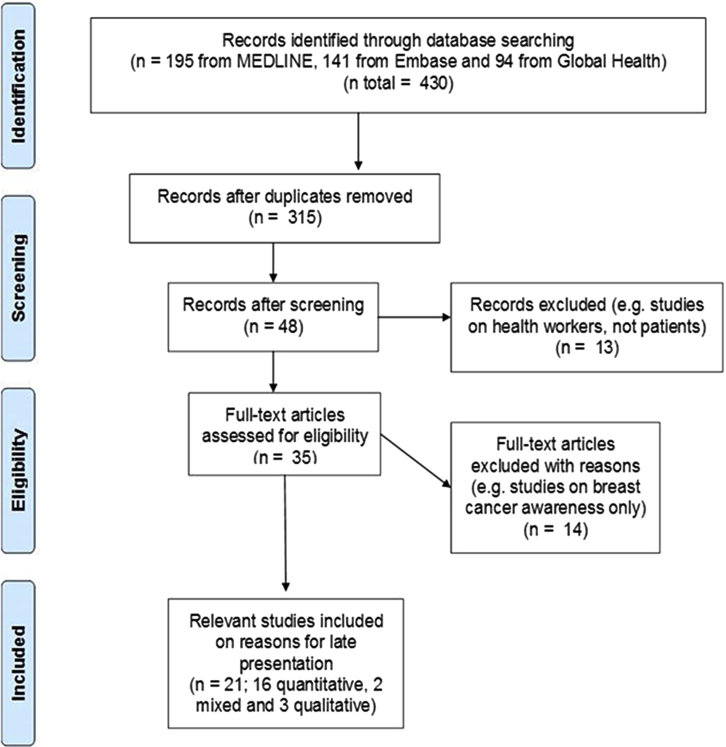
Literature search and study selection.

A total of 315 articles (after removal of duplicates) were identified through electronic searches and their titles and abstracts were screened for potential eligibility (Fig. 2). In all, 35 articles were retrieved for full-text review. Of these, only 21 were eligible for inclusion in the review: 16 quantitative studies, three qualitative studies, and two mixed studies (quantitative and qualitative).

### Study characteristics

Table 1 summarizes the main characteristics of each participating study. Of the 18 quantitative and mixed-design studies, 8 (44%) were conducted in North Africa and 10 (56%) in SSA, with their sample sizes ranging from 44 to 350. In contrast, all three qualitative studies were conducted in SSA, with sample sizes ranging from 9 to 31. All studies were hospital-based cross-sectional surveys that relied on consecutive samples of patients, except for two small qualitative studies [28], [29] that relied on convenience samples. Eligibility was restricted to women with advanced breast cancer in one study in North Africa [16] and in four studies (three quantitative [11], [21], [22] and one qualitative [28]) in SSA. The large majority of studies recruited breast cancer patients diagnosed predominantly in the years 2000–2010, but two studies in North Africa [12], [13] and two in SSA [23], [30] included patients diagnosed after 2010, whereas one study in SSA recruited patients diagnosed before 2000 [27] (Table 1). The average (mean/median) age at breast cancer diagnosis was in the 40s in the large majority of studies. Most studies involved collection of data through structured or semistructured questionnaires, usually administered by the researchers or medical staff around the time of diagnosis, but four studies were conducted retrospectively using medical records [11], [12], [21], [25]. Information on ethnicity was provided in only one study, which stated that its subjects were all black [29]. Information on tumor stage at diagnosis was available for seven (88%) studies in North Africa and nine (69%) in SSA. Among studies with stage information and whose subject eligibility was not dependent on it, the proportion of patients with late stage (III/IV) was very high (range: 46%–61% in North Africa; 76%–91% in SSA; Table 1).

**Table 1:** Main characteristics of the 21 studies included in the review

Author, year [ref no]	Country (sample size)	Hospital/clinic, location	Hospital/clinic-based catchment population∗	Recruitment				Eligibility criteria	Age (y)	Tumor characteristics					Total quality score (max. score = 30)
				Type of hospital/clinic†	Type of sample‡	Timing of§	Time period								
										First symptom(s)	Late stage‖	Size (cm)	Grade	ER status/histology	
Quantitative studies (*n* = 16)															
North Africa (*n* = 8)															
Ahmed, 2014 [11]	Sudan (*n* = 141)	National Cancer Institute, Wad Medani city	M (U: 55.6%; R: 44.4%)	T	C	Re	April 2009 to May 2010	LABC who attended the breast clinic	Md: 46 Ra: 25–71 Me: 47	n/a	LABC (IIIA: 13.2%; IIIB: 78.5%; IIIC: 8.3%)	n/a	I: 2.1% II: 20.1% III: 77.8%	ER+: 70.1% IDC: 77.1%	14
Aloulou, 2015 [12]	Morocco (*n* = 130)	Department of Radiotherapy, CHU Mohammed VI, Marrakech (public teaching hospital)	n/a	T	C	Re	Jan 2012 to Jan 2013	Histologically confirmed BC	Me: 46 Ra: 20–78	Lump: 58.5%; ulceration: 16.2%; metastasis: 13.8%; inflammation: 11.5%	T2–T4: 75%	Me: 3.5	II: 56% III: 28%	IDC: 90%	14
Benbakhta, 2015 [13]	Morocco (*n* = 200)	Department of Radiotherapy, National Institute of Oncology, Rabat	U: 74%	T	C	P	Dec 2012 to May 2013	Inclusion: all female patients with BC diagnosis treated at this institution, Moroccan nationality, provided written consent. Exclusion: those who had started neoadjuvant chemotherapy.	Me ± SD: 49.1 ± 10.7 Ra: 25–82	Breast lump: 46%	III: 43%; IV: 3%	Me: 4.1	n/a	n/a	23
El-Shinawi, 2013 [14]	Egypt (*n* = 45)	Ain Shams University Hospital Breast Clinic	M (Greater Cairo: 63%)	T	C	P	Feb 2010 to Dec 2010	Inclusion: recently diagnosed BC patients (<6 mo). Exclusion: patients unaware of their disease, recurrence disease, poor general health (289 excluded)	Md ± SD: 47 ± 10.2 Me ± SD: 48.2 ± 10.2	Painless breast mass: 57.8%; painful breast mass: 15.6%	n/a	n/a	n/a	n/a	15
Ermiah, 2012 [15]	Libya (*n* = 200)	African Oncology Institute (NOI), Sabratha	n/a	T	C	P	Jan 1, 2008 to Dec 31, 2009	Female patients with BC diagnosed at NOI	Me: 45.4 Ra: 22–75	Lump: 68%; skin changes: 15.5%; nipple discharge: 13.5%; systemic: 3.0%	III: 54%; IV: 11.5%	T1 and T2 (≤5 cm): 40%; T3 and T4: 60%	n/a	n/a	19
Landolsi, 2010 [16]	Tunisia (*n* = 160)	Dept. of Medical Oncology, Centre Hospitalier Universitaire Farhat Hached, Sousse	M (U: 37%; R: 63%)	T	C	P	Sept 1, 2005–March 31, 2006	Patients presenting with a locally advanced (T3 or T4) or a metastatic BC	Me: 48 Ra: 27–85	n/a	T3: 25%; T4: 71%; M1: 24%	Me: 6.3 cm (range: 3–15 cm)	n/a	n/a	18
Mousa, 2011 [17]	Egypt (*n* = 163)	Tanta Cancer Center, Gharbiah province (the largest cancer center in the Nile delta region)	M (U: 36.8%; R: 63%)	T	C	P	Dec 2009 to Nov 2010	Newly diagnosed BC cases	Md: 53 Me ± SD: 51.6 ± 11.5	Mass: 77.4%; pain: 7.6%; nipple discharge: 3.1%; increased breast size: 2.5%; axillary mass: 2.5%; other: 6.9%	III and IV: 60.9%	n/a	n/a	n/a	25
Stapleton, 2011 [9]	Egypt (*n* = 343)	National Cancer Institute, Cairo (*n* = 200) & Tanta Cancer Center, Gharbiah (*n* = 143)	M	T	C	P	July 2007 to Aug 2008	Inclusion criteria: females with a newly diagnosed or treated BC between July 2007 and August 2008 recruited from chemotherapy outpatient clinics. Exclusion criteria: patients aged <18 y, pregnant or lactating, previous cancer diagnosis	Me ± SD: 49.2 ± 10.9 (early stage) Me ± SD: 49.9 ± 11.0 (late stage)	n/a	Late stage: 46.1%	n/a	n/a	n/a	23
Sub-Saharan Africa (*n* = 8)															
Clegg-Lamptey, 2009 [18]	Ghana (*n* = 66)	Korle Bu Teaching Hospital	n/a	T	O	P	Sept 2007 to July 2008	Newly diagnosed BC	Md: 43 Ra: 20–84 Me: 44.8	n/a	n/a	n/a	n/a	n/a	16
Ezeome, 2010 [19]	Nigeria (*n* = 162)	University of Nigeria Teaching Hospital Enugu	n/a	T	C	P	June 1999 to June 2001 and April 2003 to May 2005	BC patients managed at the Surgical Oncology unit at the University of Nigeria Teaching Hospital Enugu who provided consent	Md: 45 Ra: 21–77 Me: 45.7	n/a	III: 40.8%; IV: 37.5%	n/a	n/a	n/a	23
Ibrahim, 2012 [20]	Nigeria (*n* = 201)	Lagos State University Teaching Hospital	U	T	C	P	Jan 2009 to Dec 2010	All female BC patients referred to one of the general surgery outpatient clinics of Lagos State University Teaching Hospital	Me: 49.82 (SD: 13.59) Ra: 23–104	n/a	III: 62.7%; IV: 16.4%	n/a	n/a	n/a	23
Marcus, 2013 [21]	South Africa (*n* = 103)	Sebokeng Hospital, Gauteng	U	Level 2 public regional hospital	C	Re	Jan 2007 to Dec 2010	All patients presenting at the breast clinic with advanced BC (IIB or higher)	Me: 59 Ra: 34–83	Breast lump: 84.5%; axillary node abnormal: 19.4%; abscess/ulcers: 7.8%; nipple discharge: 6.8%; pain: 4.9% (not mutually exclusive)	III–IV: 95.1%	n/a	n/a	n/a	13
Otieno, 2010 [22]	Kenya (*n* = 166; 98.8% females)	Kenyatta National Hospital	M	T	C	P	Oct 1, 2003 to 31 March, 2006	Inclusion: all (male and female) patients who attended the breast clinic or were admitted to the three surgical wards with advanced BC (stages III/IV). Exclusions: patients with treated or recurrent BC	Me: 47 Ra: 17–88	Breast lump: 87.3%	III/IV: 100%	n/a	n/a	n/a	15
Pace, 2015 [23]	Rwanda (*n* = 144)	Butaro and Rwinkwavu rural hospitals	R	S or T (n/a)	C	P	Nov 2012 to Feb 2014	Inclusion: women aged ≥21 y with pathologically confirmed BC. Exclusions: women diagnosed elsewhere >6 mo without initial staging	Md: 49	Breast pain: 59%	III: 52%; IV: 24%	n/a	n/a	n/a	25
Price, 2012 [24]	Cameroon (*n* = 50 BC cases; includes other cancers)	Yaounde General Hospital—the only one in the country to offer chemotherapy	M	T	C	P	July 13, 2010 to Aug 12, 2010	Patients aged ≥18 y with primary invasive BC (98% with histological confirmation) and who received chemotherapy; 96% female	Me: 46 Ra: 29–75	n/a	n/a	n/a	n/a	n/a	20
Toure, 2013 [25]	Cote d'Ivoire (*n* = 350)	University Hospital of Treichville, Abidjan	M	T	C	Re	Jan 2008 to Dec 2011	Patients with a histologically confirmed adenocarcinoma of the breast	Me: 42 Ra: 18–81	Breast lump: 6%; inflammation: 54%; ulcer: 18%; nipple blood discharge: 8%; metastases: 14%	III: 76.3%; IV: 14.3%	n/a	n/a	Adenocarcinoma: 100%	19
Quantitative and qualitative studies (*n* = 2)															
Dye, 2010 [26]	Ethiopia (*n* = 69; 98.1% females)	Tikur Anbessa Hospital	M	T	C	P	2008 (1 mo only)	Randomly selected female and male BC patients seen at Tikur Anbessa Hospital over the span of 1 mo (similar characteristics to the total population). Patients or their families were interviewed.	Me: 44.5	n/a	n/a	n/a	n/a	n/a	10
Ly, 2002 [27]	Mali (*n* = 44; 43 females)	Hôpital du Point-G, Bamako	M	T	C	P	Sep 15, 1998 to Aug 15, 2000	Newly diagnosed and histologically confirmed BC patients (male and female) seen at the hematology/oncology service	Me (SD): 46 ± 19.5 Ra: 25–80	Breast lump: 39%; breast pain: 39%; pruritus (itching): 12%; nipple blood discharge: 6.8%; ulcer: 4.5%	III: 40.9%; IV: 45.5%	n/a	n/a	n/a	7
Qualitative studies (*n* = 3)															
Ekortarl, 2007 [28]	Cameroon (*n* = 9 BC cases; 11 subjects with other types of cancer)	Yaounde General Hospital	M	T	O	P	n/a	Cancer patients who presented with advanced disease or who reappeared at an advanced stage after having abandoned treatment at the oncology division	Ra: 34–63	n/a	Advanced BC: 100%	n/a	n/a	n/a	n/a
Mbuka-Ongona, 2012 [29]	Botswana (*n* = 11)	Princess Marina Hospital, Gaborone (the only hospital in the country with oncology services)	M	T	O	P	2007	Inclusion: all female adult BC patients seen and managed at Princess Marina Hospital. Exclusions: aged <18 y; too ill; or mentally incapacitated	Me: 54 Ra: 37–76	Most common: painless lump; second most common: bloody nipple discharge	Majority stage III	n/a	n/a	n/a	n/a
Pruitt, 2015 [30]	Nigeria (*n* = 31)	University College Hospital Ibadan	M	T	C	P	July 2011	All female BC patients seen in the radiotherapy and surgery clinics, aged ≥18 y, regardless of ethnicity, language, or stage.	Md: 51 Ra: 28–80	n/a	n/a	n/a	n/a	n/a	n/a

BC = breast cancer; BSE = breast self-examination; CBE = clinical breast examination; CHU = Centre Hospitalier Universitaire; ER = estrogen receptor; IDC = invasive ductal carcinoma; IQR = interquartile range; LABC = locally advanced breast cancer; Md = median; Me = mean; n/a = not reported in the original publication; Ra = range.

∗ Population-based: urban (U), rural (R), mixed (M) area, or not reported (n/a).

† Primary (P), secondary (S), or tertiary (T) hospital/clinic.

‡ Opportunistic (O) or consecutive (C) sample of patients.

§ Patients recruited prospectively (P) or retrospectively (Re).

‖ Stages III–IV (note: T2 can be staged as III A).

Quality scores were low for most quantitative studies (Table 1) albeit slightly higher for those from North Africa (median = 18.5; range: 14–25) than for those from SSA (median = 17.5; range: 7–25). Similarly, the quality of the qualitative and mixed-design studies varied substantially, with three studies presenting more in-depth qualitative results [26], [29], [30].

### Delays in presentation and diagnosis

**Fig. 3:**
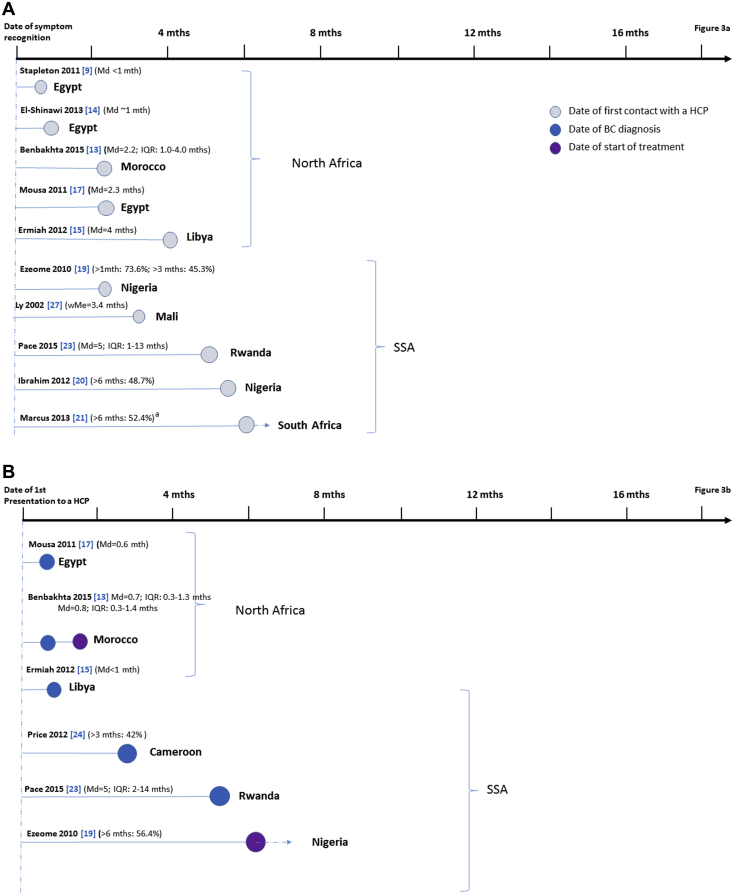

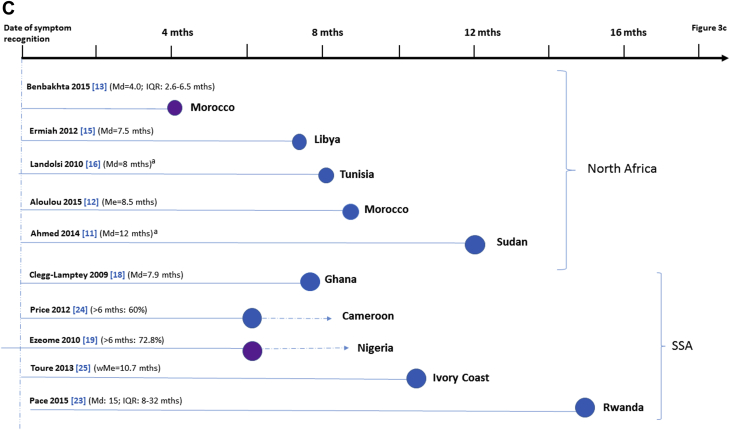
Study-specific delays in breast cancer: (A) from symptom recognition by the patient to her presentation to the first health care provider; (B) from presentation to breast cancer diagnosis or start of cancer treatment; and (C) from symptom recognition to diagnosis or start of treatment. ^a^Study eligibility restricted to advanced BC. See Table 2 for more detailed information on study-specific estimates of delay. A dashed line indicates that the delay estimate shown in the figure is an underestimation of the median value (the latter could not be calculated from the data provided in the original article). No delay estimates for Otieno et al. [22] are shown because average time from symptoms to diagnosis could not be estimated (>3 months for 73% of patients—all with advanced BC—with no further information provided; see Table 1, Table 2). BC = breast cancer; HCP = health care provider; IQR = interquartile range; Md = median; Me = mean; SSA = sub-Saharan Africa; wMe = weighted mean.

The time interval between symptom recognition by the woman to presentation, that is, to first visit to a health care provider, varied substantially across studies but, overall, it was shorter in North Africa than in SSA (Table 2; Fig. 3A). Of the five North African studies that reported on presentation delays, most yielded median estimates of less than 2.5 months; the only exception was a study in Libya [15] with a median presentation time of 4 months. Of the five studies in SSA that provided estimates of time from symptom recognition to presentation, only one [19] reported a median time of less than 2.5 months, with the remaining reporting average times ranging from 3.4 months in Mali [27] to greater than 6 months in South Africa [21].

**Table 2:** Time from recognition of potential symptoms of breast cancer to presentation to the first health care provider, diagnosis and start of treatment, and number of health care providers visited

Author, year [ref no]	Country (sample size)	Time from			No. of health care providers visited before visit to the one where diagnosis was made
		Symptom recognition to presentation	Presentation to diagnosis	Diagnosis to start of treatment	
North Africa					
Ahmed, 2014 [11]∗	Sudan (*n* = 141)	Md: 12 mo; Ra: 2–108 mo		n/a	n/a
Aloulou, 2015 [12]	Morocco (*n* = 130)	Me: 8.47 mo; > 6 mo: 63.1%		n/a	n/a
Benbakhta, 2015 [13]	Morocco (*n* = 200)	Md: 65 days (=2.17 mo); IQR: 31–121 days; Ra: 3–579 days	Md: 20 days (=0.67 mo); IQR: 10–40 days; Ra: 1–433 days	Md: 25 days (=0.83 mo); IQR: 9–42 days; Ra: 0–368 days	n/a
			Md: 50 days (=1.67 mo); IQR: 29, 77 days; Ra: 5–535 days		
		Md: 120 days (4.0 mo); IQR: 81–202 days; Ra: 14–860 days			
El-Shinawi, 2013 [14]	Morocco (*n* = 45)	<1 mo: 46.7% 1 to <6 mo: 37.8% 6 to <12 mo: 0% >12 mo: 15.6%	n/a	n/a	n/a
Ermiah, 2012 [15]	Libya (*n* = 200)	Md: 4 mo (max. 24) <3 mo: 46% 3–6 mo: 14% >6 mo: 40%	Md: < 1 mo <1 mo: 84.5% 1–6 mo: 4.5% >6 mo: 11.0%	n/a	n/a
		Md: 7.5 mo (max. 25 mo) <3 mo: 30% 3–6 mo: 14% >6 mo: 56%			
Landolsi, 2010 [16]∗	Tunisia (*n* = 160)	Mean: 11.6 mo; Md: 8 mo		n/a	n/a
Mousa, 2011 [17]	Egypt (*n* = 163)	Me: 6.2 mo; Md: 2.3 mo	Presentation to arrival at TCC: Me: 6.8 wk; Md: 2.5 wk	n/a	Me: 1.5; Ra: 0–4 (does not mention traditional or spiritual healers)
Stapleton, 2011 [9]	Egypt (*n* = 343)	Md: <1 mo	n/a	n/a	n/a
Sub-Saharan Africa					
Clegg-Lamptey, 2009 [18]	Ghana (*n* = 66)	Me: 46 wk (=10.7 mo) Md: 34 wk (=7.9 mo) Ra: 1 wk, 5 y		n/a	Previous medical consultation: 39.4%
Ezeome, 2010 [19]	Nigeria (*n* = 162)	<1 mo: 26.4% 1–3 mo: 28.3% >3 to 6 mo: 17.6% >6 mo: 27.7%	<1 mo: 17% 1–3 mo: 10.6% >3 to 6 mo: 16% >6 mo: 56.4%		n/a
		<1 mo: 5.6% 1–3 mo: 4.3% >3 to 6 mo: 17.3% >6 mo: 72.8%			
Ibrahim, 2012 [20]	Nigeria (*n* = 201)	Me (SD): 12.12 (5.18) mo Ra: 1 wk to 96 mo <1 mo: 4.5% 1–3 mo: 13.9% >3 to 6 mo: 32.8% >6 to 12 mo: 30.8% >12 mo: 17.9%	n/a	n/a	n/a
Marcus, 2013 [21]∗	South Africa (*n* = 103)	<3 mo: 17.5% 3–6 mo: 30.1% >6 mo: 52.4%	n/a	n/a	n/a
Otieno, 2010 [22]∗	Kenya (*n* = 166; 98.8% females)	From first symptoms to presentation at Kenyatta National Hospital (late stage only) <30 d: 6.62% 31–90 d: 20.4% >90 d: 73.08%		n/a	n/a
Pace, 2015 [23]	Rwanda (*n* = 144)	Md: 5 mo (IQR: 1–13)	Md: 5 mo (IQR: 2–14)	n/a	<5 HCP visits: 44% ≥5 HCP visits: 56% (does not mention traditional or spiritual healers)
		Md: 15 mo (IQR: 8–32)			
Price, 2012 [24]	Cameroon (*n* = 50)	n/a	>3 mo: 42% >6 mo: 32%	n/a	Consulted ≥4 HCP: 46% (including traditional and spiritual healers)
		>6 mo: 60%			
Toure, 2013 [25]	Cote d'Ivoire (*n* = 350)	<6 mo: 9.1% 6–10 mo: 12% 10–14 mo: 78.9% Weighted mean: 10.7 mo		n/a	n/a
Quantitative and qualitative studies					
Dye, 2010 [26]	Ethiopia (*n* = 69; 98.1% females)	n/a	n/a	n/a	>2 HCP visits: 73.2% (including traditional or spiritual healers)
Ly, 2002 [27]	Mali (*n* = 44; 43 females)	1–12 wk (=2.8 mo): 63.6% 13 (=3.0 mo) to 48 wk (=11.2 mo): 36.4% Weighted mean: 3.4 mo	n/a	n/a	>3 HCP: 50% (only conventional HCP included)
		From symptoms to first appointment at the study (diagnostic) hospital: Ra: 8 wk (=1.87 mo) to 72 wk (=16.8 mo)			
Qualitative studies					
Ekortarl, 2007 [28]	Cameroon (*n* = 9 BC cases; 11 subjects with other types of cancer)	n/a	n/a	n/a	n/a
Mbuka-Ongona, 2012 [29]	Botswana (*n* = 11)	Time from first symptom to presentation at study hospital (PMH): Me: 3 y; Ra: 1–10 y		n/a	n/a
Pruitt, 2015 [30]	Nigeria (*n* = 31)	n/a		n/a	n/a

BC = breast cancer; CI = confidence interval; HCP = health care provider; IQR = interquartile range; Md = median; Me = mean; n/a = not reported in the original publication; Ra = range; TCC = Tanca Cancer Center.

∗ Study recruited only patients with advanced breast cancer (see Table 1).

Fewer studies in North Africa [13], [15], [17] and in SSA [19], [23], [24] gave estimates of the time between presentation and diagnosis, or between diagnosis and start of treatment. Nevertheless, the length of these intervals tended to be shorter than the length of the corresponding intervals between symptom recognition and presentation in North Africa (all <1 month) but not in SSA (Fig. 3B).

Five North African studies provided median estimates of the total delay from symptom recognition to date of breast cancer diagnosis or start of treatment (Fig. 3C). Two of these studies recruited only advanced breast cancer cases with average total delays of 8 [16] and 12 months [11]. Median estimates of the total delay from symptom recognition to diagnosis for the remaining three studies ranged from 4 [13] to 8.5 months [12]. Five SSA studies provided average times from presentation to diagnosis or start of treatment (Fig. 3C), with their estimates ranging from 7.9 months in Ghana [18] to 15 months in Rwanda [23]; median delays were known to be greater than 6 months for two studies [19], [24], but their exact values could not be estimated. In addition, a small qualitative study (*n* = 11) in Botswana reported a median time from first symptom(s) to presentation at the hospital where the diagnosis was finally made of 3 years [29].

The number of health care providers visited before the one where the diagnosis was made was reported by only one study in North Africa [17] and four in SSA [23], [24], [26], [27], with estimates ranging from a median of 1.5 in Egypt [17] to greater than 5 in Rwanda [23]; however, these estimates are not entirely comparable because traditional and religious healers were included in two of these studies [24], [26].

A few studies examined whether delays were associated with late stage (III/IV) at diagnosis. The study by Benbakhta et al. [13] in Morocco reported a 6.81-fold (95% confidence interval [CI], 3.65–12.7) increase in the odds of late stage among patients who delayed presentation by greater than 64 days relative to those who presented less than or equal to 64 days of symptom recognition. Similarly, the odds of late stage among patients who experienced a diagnostic delay of greater than or equal to 50 days were 1.84 (95% CI, 1.05–3.23) times higher than among those diagnosed less than 49 days of their first presentation to a health care provider [13]. The study by Mousa et al. [17] in Egypt also reported an association between late stage and delays in presentation greater than 3 months (crude odds ratio: 1.99; 95% CI, 1.01–1.99) but not with delays in diagnosis greater than 2 weeks. In Rwanda, late stage was positively associated with both presentation (median [range] in months: 2 [1–12] for stages I/II, 5 [1–13] for stage III, and 9 [3–18] for stage IV; *P* = .09) and diagnostic delays (4 [2–13] months for stage I/II, 4 [2–10] for stage III, and 11 [5–28] for stage IV; *P* = .005) [23].

### Factors associated with delays

Appendix C summarizes the reasons most commonly reported by the quantitative studies in the review for late presentation to the first health care provider. They fell into the following categories: (1) socioeconomic factors such as low educational level; (2) lack of breast cancer awareness and poor knowledge of early-detection methods (e.g., breast self-examination); (3) type of initial symptoms: painless, not taken seriously, or hoping they would resolve soon; (4) fear of the disease, its treatment (e.g., mastectomy) or death, or of being a burden to the family; (5) belief in traditional medicine or spiritual cures; (6) financial constraints; and (7) poor access to health care (e.g., living too far away from a health care provider; lack of transportation). Benbakhta et al. [13] found in mutually adjusted analysis that a delay in presentation of greater than or equal to 2.2 months in Morocco was positively associated with low socioeconomic conditions (e.g., living in a rural area, being illiterate, being a housewife [vs. being employed], and having low socioeconomic level) and lack of breast cancer awareness (e.g., negative family history of cancer, no knowledge of breast self-examination) (Appendix C). In contrast, Mousa et al. [17] found no association between delay in presentation greater than 3 months in Egypt and a woman's socioeconomic characteristics or type of symptoms before or after adjustment for potential confounders. In South Africa, Marcus et al. [21] found in mutually adjusted analysis the positive associations with late presentation (>6 vs. 3–6 months) with increasing age and a previous cancer diagnosis but not with educational level, marital status, or being employed/unemployed. A mutually adjusted analysis of data from a study in Rwanda [23] revealed a four-fold to five-fold increase in the odds of late presentation (≥6 months) for patients with low or no education and for those who visited a traditional healer first but no independent associations with other socioeconomic factors, breast cancer awareness, symptom, or health services–related variables (Appendix C). Overall, the findings from the qualitative studies supported the evidence from the quantitative studies [26], [28], [29], [30] (Appendix C).

The reasons given by the patients for delays between presentation and diagnosis, or start of treatment, included patient-mediated factors (e.g., socioeconomic factors, type of symptoms, having tried traditional treatments first, financial problems, fear of the disease and/or its treatment, and denial) and health care provider–mediated factors (e.g., travel time to health care provider, the number and type of health care providers contacted before diagnosis, delayed referrals or nonreferrals, misdiagnosis, wrong advice or false reassurances, delays in obtaining diagnostic confirmation, and in starting treatment) (Appendix C). The study in Morocco by Benbakhta et al. [13] found in mutually adjusted analyses that a delay greater than 1.7 months between presentation and start of treatment was associated with older age, illiteracy, low socioeconomic level, distance to health care provider greater than or equal to 100 km, and greater than or equal to 3 consultations before the diagnostic one. Mousa et al. [17] in Egypt showed that after adjustment for potential confounders, the odds of a delay greater than 2 weeks from the first medical consultation to arrival at the diagnostic center were not associated with the patient's age, socioeconomic conditions, or type of symptoms but were strongly associated with the type of the first health care provider visited and the navigation pathway followed by the patient (Appendix C). In Rwanda, Pace et al. [23] found in mutually adjusted analyses a 2.69 (95% CI, 1.24, 5.84) higher odds of a delay greater than or equal to 6 months for patients who visited five or more health care facilities before diagnosis but no associations with the patient's socioeconomic conditions, reproductive history, or type of symptoms. In the qualitative studies (Appendix C), some women reported poor clinical practices (e.g., inadequate diagnosis by general doctors [28], hospital strikes [30], or having sought alternative care after receiving the diagnosis).

## Discussion

To our knowledge, this is the first systematic review of studies that reported on delays in a woman's breast cancer journey in Africa. Its findings highlighted three main issues. First, there is a paucity of published data on delays in the presentation and diagnosis of the most common female cancer in Africa [2]. The systematic review identified only 21 published studies over the 16-year period (January 2000 to May 2016), comprising only 2788 breast cancer patients from across the continent (1382 from North Africa; 1406 from SSA). Second, the findings revealed marked delays in presentation and diagnosis of breast cancer patients in both North Africa and SSA. Third, the reported reasons for such delays were complex and included both patient-mediated and health system–mediated factors; however, the relative importance of these two types of factors varied from setting to setting.

There is strong evidence that a delay from symptom recognition to diagnosis of more than 3 months is associated with later stage at presentation and poorer survival [6]. This review revealed substantially longer delays in both North Africa and SSA, with reported average times from symptoms recognition to diagnosis between 4 and 15 months. These estimates are in line with those observed in other low- and middle-income countries (LMICs) (e.g., 7.6 months in Brazil [31], 5.5 months in Malaysia [32]) but much higher than those observed in HICs (e.g., 34 days in France [33], 48 days in the United States [34]). The very long time intervals from symptom recognition to diagnosis in Africa resulted from delays in both presentation and diagnosis. All studies in this review, with the exception of two [9], [14], reported average presentation intervals between 2.2 months and greater than 6 months, much longer than those observed in HICs (e.g., 9 days in the United Kingdom [35]; 16 days in Germany [36]). Similarly, reported diagnostic intervals in Africa were much longer than those found in HICs (e.g., from 10 to 42 days in France [33], Germany [37], and the United States [34]) but similar to what has been described for other LMICs (e.g., median of 5 months in Brazil [31], Colombia [38], and Mexico [39]).

As we had hypothesized in our conceptual model, delays in presentation in Africa were found to be associated not only with patient-mediated factors (e.g., low educational level, poor breast cancer awareness, use of alternative care medicine) but also with health service–mediated factors (e.g., distance to the nearest health care center). These results are similar to those from previous studies—for example, being unaware of the warning signs or tests for breast cancer [5], patients only seeking conventional care when traditional treatment has failed [40], or inability to afford the costs of treatment [41]. Similarly, delays in diagnosis in Africa were influenced by both patient-mediated factors (e.g., low educational level, financial problems) and health system–mediated factors (e.g., type of first health care provider visited, number of providers visited before diagnosis, type of navigation pathway followed before reaching the diagnostic center). A high number of referrals make the patient's journey through the health system longer resulting in a more advanced tumor stage at diagnosis; however, it is also conceivable that a low number of referrals might reflect a more aggressive tumor, or a longer time interval before presentation to the first health care provider, and thus a more advanced tumor that was easily identified by the physician. Of note, however, is the fact that none of the articles directly examined health system factors, for example, through interviews with health care providers, relying instead on patients' reports.

### Strengths and limitations of the review

Major strengths of this review include the systematic search strategy used to identify eligible English and non-English publications and the use of standardized methods for data extraction and synthesis. The review also has weaknesses. Its representativeness may have been compromised by several factors. First, publication bias cannot be excluded as gray literature was not included in this review. Second, the review included studies from only 4 of 7 North African countries and 11 of 51 SSA countries, albeit the latter comprised studies from all four SSA regions (i.e., from Eastern, Western, Southern, and Middle Africa). Third, none of the studies in the review were population based; they were all hospital based, predominantly from tertiary hospitals as these are the only ones in most African countries to have appropriate cancer diagnostic and treatment facilities. However, such studies excluded, by design, the large number of patients who never reach tertiary hospitals, some of whom are never diagnosed. Hence, the included patients who reached tertiary facilities are unlikely to be a representative sample of all breast cancer patients in Africa.

The methodological quality of most articles was low. In particular, measurement errors may have affected the validity of the review's findings as although most of the studies recruited women prospectively, patients were asked to remember the time from first symptom(s) to presentation, and this might have introduced recall errors and even biases. Little detail was provided in the original articles on the specific instruments used to collect information and the methods used to estimate times to presentation, diagnosis, and treatment, including on the way questions to patients on time intervals were formulated and on how relevant time-related events (e.g., dates of contact with a first health care provider, breast cancer diagnosis, and start of treatment) were defined. Between-study differences in these methodological issues may have affected their comparability. When questioned about the reasons for delays, patients might have been reluctant to admit less orthodox behaviors such as the use of traditional medicine. Reassuringly, however, the studies that examined associations between self-reported delays and late stage at diagnosis showed, as expected, strong positive associations. Many studies had relatively small sample sizes, and thus, their ability to precisely quantify delays and their power to detect associations were limited. There were large variations across studies in the way data were analyzed (e.g., only a few quantitative studies attempted to control for confounders; none of the qualitative studies conducted theoretical analyses) and summary findings presented, hampering between-study comparisons, and precluding the conduct of meta-analyses.

## Conclusions

Several studies in Africa have shown that early-stage breast cancer is associated with better survival than late-stage disease [42], [43], consistent with early diagnosis and treatment being associated with reductions in mortality from this disease in the region. The long presentation and diagnostic delays identified by this review indicate that there is considerable potential to introduce interventions aimed at shrinking the time intervals between symptom recognition and diagnosis. Mammography screening is often advocated as the best intervention to improving early diagnosis of breast cancer, but the findings from this review strongly argue against adopting such an approach in African settings. Screening can only reduce breast cancer mortality if women with suspicious screen-detected lesions have access to appropriate diagnosis and treatment. Despite the limitations of the existing data, and the high heterogeneity across African settings, the long diagnostic delays highlighted by the review indicate that the addition of women with asymptomatic screen-detected tumors would place significant additional burden on most, already overstretched, health care systems in the region. Instead, downward-stage migration of symptomatic breast cancer should be the priority in most settings as recommended by the Breast Health Global Initiative and the Breast Cancer Initiative 2.5 [44]. Achieving this would require increased breast cancer awareness of the population, enhanced ability of primary and secondary health care professionals to diagnose breast cancer, as well as clear patient navigation pathways to facilitate timely referral and admission of patients to tertiary care services for early care. The introduction of such an approach in other LMICs has demonstrated that downward-stage migration of breast cancer is achievable in the absence of screening [45].

## Figures and Tables

**Appendix A:** Examples of the search string used in MEDLINE

1. (breast OR mammary) ADJ3 (neoplasm* OR cancer* OR tumo?r* OR carcinoma)
2. exp Breast Neoplasms
3. 1 OR 2
4. (delay* OR late OR poor) ADJ1 (presentation OR attendance OR diagnosis OR stage OR detection OR prognosis)
5. exp Delayed Diagnosis
6. exp Prognosis
7. exp Early Diagnosis
8. 4 OR 5 OR 6 OR 7
9. (determinant* OR factor* OR reason* OR barrier* OR attitude* OR belie* OR awareness OR knowledge OR fear* OR cultur* OR perception*)
10. (uptake OR utilization OR access OR accept* OR intent* OR distance OR transport* visit* OR presentation*) ADJ3 (health care centre OR hospital OR clinic OR health service OR doctor OR physician OR mammogram* OR screening OR exam*)
11. exp “Behavior and Behavior Mechanisms”
12. exp Attitude to Health
13. exp Socioeconomic Factors
14. exp Health Status Disparities
15. exp Communication Barriers
16. OR/9-15
17. Africa OR Algeria OR Angola OR Benin OR Botswana OR Burkina Faso OR Burundi OR Cameroon OR Cape Verde OR Central African Republic OR Chad OR Democratic Republic of the Congo OR Djibouti OR Egypt OR Equatorial Guinea OR Eritrea OR Ethiopia OR Gabon OR Gambia OR Ghana OR Guinea-Bissau OR Guinea OR Ivory Coast OR Kenya OR Lesotho OR Liberia OR Libya OR Madagascar OR Malawi OR Mali OR Mauritania OR Mauritius OR Morocco OR Mozambique OR Namibia OR Niger OR Nigeria OR Republic of Congo OR Rwanda OR Senegal OR Sierra Leone OR Somalia OR South Africa OR South Sudan OR Sudan OR Swaziland OR Tanzania OR Togo OR Tunisia OR Uganda OR Zambia OR Zimbabwe
18. exp Africa
19. 17 OR 18
20. 3 AND 8 AND 16 AND 19

**Appendix C:** Factors associated with delayed presentation and delayed diagnosis or start of treatment of breast cancer in Africa: summary of the findings reported by the studies included in the review

Author, year [ref no] (country)	Factors associated with delay between symptoms recognition and first visit to an HCP		Factors associated with delay between first visit to an HCP and BC diagnosis or start of treatment	
	Crude	Adjusted	Crude	Adjusted
North Africa				
Ahmed, 2014 [11]† (Sudan)	**Factors associated with late presentation in patients with locally advanced breast cancer (%)** Lack of education: 39.5% Financial aspects: 28.6% Use of traditional medicine: 13.8% Limited access to medical care: 9% Ignorance: 6.9% Fear of being a burden to relatives: 2.7%		n/a	
Aloulou, 2015 [12] (Morocco)	**Reasons for delays from symptoms recognition to diagnosis:** Fear of cancer and/or treatment: 4%; Financial problems: 40%; Tried traditional treatments: 20% *Health services:* Distance from health center: 23%; Wrong diagnosis: 6%; Inadequate medical care: 7%			
Benbakhta, 2015 [13] (Morocco)	**Delay from symptoms recognition to presentation >2.2 mo: OR (95% CI)** *Socioeconomic:* Aged >65 versus <45 y: 1.68 (0.64, 4.38) Rural versus urban area of residence: **4.62 (2.24, 9.52)** Illiteracy versus secondary/university: **4.56 (2.26, 9.18)** Employed versus housewife: **0.23 (0.13, 0.57)** Low versus mid socioeconomic level: **8.55 (3.16, 23.17)** >5 people in household: **2.05 (1.14, 3.69)** *BC awareness:* No knowledge versus knowledge of BSE: **17.88 (8.74, 36.56)** Positive versus negative family history: **2.51 (1.23, 5.13)** *Type of symptoms:* Presence of typical versus atypical symptoms: 0.75 (0.33, 1.67) *Health services related:* Distance from HCP of presentation ≥100 versus <100 km: **8.62 (1.01, 67.14)**	Delay from symptoms recognition to presentation >2.2 mo: OR* (95% CI) *Socioeconomic:* Rural versus urban area of residence: **3.00 (1.24, 7.23)** Illiteracy versus secondary/university: **4.90 (2.50, 6.30)** Employed versus housewife: **0.1 (0.03, 0.47)** Low versus mid socioeconomic level: **7.60 (2.24, 25.77)** *BC awareness:* No knowledge versus knowledge of BSE: **11.51 (5.18, 25.57)** Negative versus positive family history: 2.11 (1.10, 4.16) *Mutually adjusted	**Delay between presentation and start of treatment >1.7 mo: OR (95% CI)** *Socioeconomic:* Aged >65 versus <45 y: **1.94 (1.36, 2.40)** Rural versus urban area of residence: **2.10 (1.18, 4.40)** Illiteracy versus secondary/university: **2.70 (1.38, 5.27)** Low versus mid socioeconomic level: **2.61 (1.20, 23.17)** *Health services:* Distance to HCP of diagnosis >100 versus <100 km: **2.46 (1.26, 5.20)** ≥3 versus <3 consultations before diagnostic one: **11.44 (4.83, 27.08)**	Delay between presentation and start of treatment ≥1.7 mo: OR* (95% CI) *Socioeconomic:* Aged >65 versus <45 y: **2.51 (1.50, 11.42)** Illiteracy versus secondary/university: **1.40 (1.12, 6.50)** Low versus mid socioeconomic level: **2.59 (1.04, 6.50)** *Health services:* Distance to HCP of diagnosis ≥100 versus 100 km: **2.58 (1.12, 3.56)** ≥3 versus <3 consultations before diagnostic one: 11.27 (4.12, 28.34) *Mutually adjusted
El-Shinawi, 2013 [14] (Egypt)	**Delay from symptoms recognition to presentation to an HCP** *BC awareness:* Higher awareness of BSE associated with less delay in seeking medical advice (2.9 + 2.3 mo) relative to low awareness (15.5 + 22.6 mo) (*P* = .04)	n/a	n/a	n/a
Ermiah, 2012 [15] (Lybia)	**Delay from symptom recognition to diagnosis >3 mo** *Socioeconomic:* Aged ≥50 versus <50 y: **64%** versus **51% (*P* = .033)** Single versus married: 52% versus 56% (*P* = .6) Housewife versus employed: 61% versus 48% (*P* = .09) Illiteracy versus literacy: **69%** versus **38% (*P* = .009)** *Reproductive:* Postmenopausal versus premenopausal: 64% versus 50% (*P* = .05) No versus breastfeeding: 38% versus 58.6% (*P* = .09) OC use >5 y versus <5 y or no use: **86%** versus **53% (*P* = .04)** *BC awareness:* Positive versus negative family history: 45% versus 57% (*P* = .3) Positive versus negative history of benign breast disease: **73%** versus **52% (*P* = .03)** Knowledge of BSE versus no knowledge: **0%** versus **58% (*P* < .0001)** *Type of symptoms:* Initial symptom being a lump versus being other symptoms: **41%** versus **86% (*P* < .0001)**			
Landolsi, 2010 [16]† (Tunisia)	**Delay from symptoms recognition to presentation at study setting, i.e., to diagnosis** *93% delay related to personal reasons:* Not aware of disease: 35% Not having practiced BSE: 23.5% Fear of cancer and/or treatment: 14% Financial problems: 14% Others: 13.5% *24% delay related to health services:* Wrong reassurance: 47.5% Misdiagnosis: 18%			
Mousa, 2011 [17] (Egypt)	**Delay from symptoms recognition to first medical consultation >3 mo: OR (95% CI)** *Socioeconomic:* Aged ≥50 versus < 50 y: 1.1; 95% CI: 0.6, 2.1 Urban versus rural residence: 1.3; 95% CI: 0.7, 2.6 ≥Bachelor versus < bachelor education: 0.6 (0.3, 1.2) *Type of symptoms:* Breast mass versus other first symptom: 2.1 (0.9, 4.8)	Delay from symptoms recognition to first medical consultation >3 mo: OR* (95% CI) *Socioeconomic:* Aged ≥50 versus < 50 y: 0.9 (0.4, 1.9) Urban versus rural residence: 1.4 (0.7, 2.9) ≥Bachelor versus <bachelor education: 0.6 (0.3, 1.2) *Type of symptoms:* Breast mass versus other first symptom: 2.1 (0.9, 4.8) *Adjusted for age, residential status, and education	**Delay from first medical consultation to arrival at TTC >2 wk: OR (95% CI)** *Socioeconomic:* Aged ≥50 versus < 50 y: 0.6 (0.3, 1.2) Urban versus rural residence: 0.8 (0.4, 1.5) ≥Bachelor versus <bachelor education: 1.2 (0.7, 2.3) *Type of symptoms:* Breast mass versus other first symptom: 0.8 (0.4, 1.8) *Health services–related:* First health care provider versus TCC: Primary care: **11.0 (2.9, 41.7)** Gynecologist: **9.0 (1.6, 52.3)** Medical oncologist: **5.6 (1.0, 30.9)** General surgeon: **5.5 (1.7, 18.0)** Surgical oncologist: 3.0 (0.7, 13.4) Other: **12.0 (2.2, 66.5)** Navigation pathway versus directly to TCC: General surgeon → Surgical oncologist→ TCC: **29.3 (4.6, 184.4)** General surgeon → Medical oncologist→ TCC: 6.0 (0.9, 38.1) Primary care→ Others→ TCC: **19.5 (3.7, 102.4)**	Delay from first medical consultation to arrival at TTC >2 wk: OR* (95% CI) *Socioeconomic:* Aged ≥50 versus < 50 y: 0.6 (0.3, 1.4) Urban versus rural residence: 1.1 (0.5, 2.3) ≥Bachelor versus <bachelor education: 1.3 (0.5, 2.9) *Type of symptoms:* Breast mass versus other first symptom: 1.3 (0.6, 3.1) *Health services–related:* First HCP versus TCC: Primary care: **12.2 (2.9, 51.0)** Gynecologist: **8.6 (1.4, 53.4)** Medical oncologist: **8.3 (1.3, 55.0)** General surgeon**: 7.6 (2.1, 27.6)** Surgical oncologist: 3.4 (0.7, 16.0) Other: **11.0 (1.9, 63.3)** Navigation pathway versus directly to TCC: General surgeon → Surgical oncologist → TCC: **35.4 (5.3, 237.5)** General surgeon → Medical oncologist→ TCC: **8.1 (1.0, 62.2)** Primary care→ Others → TCC: 23.2 (4.0, 134.5) *Adjusted for age, residential status, education level, tumor stage, and first symptom
Stapleton, 2011 [9] (Egypt)	**Late versus early stage at diagnosis: Mutually adjusted OR (95% CI)** >33 wk versus ≤33 delay in seeking treatment: 1.57 (0.76, 3.23) *Financial and other constraints* Social, financial, and time constrains versus no delay: 1.72 (0.86, 3.46) *Type of symptoms:* No pain versus no delay: **2.68 (1.18, 6.08)** *BC awareness:* Knowledge of BSE versus no knowledge: **0.24 (0.06, 0.94)** Previous CBE versus no previous CBE: 1.00 (0.28, 3.62) Previous mammogram versus no previous mammogram: 2.17 (0.48, 9.72) *Health services related:* Site of treatment NCI-Cairo versus TCC: **5.05 (1.30, 19.70)** Visited versus not visited a second provider: 0.72 (0.30, 1.74) First diagnosed versus not first diagnosed as BC: 0.99 (0.52, 1.89) Referral versus no referral: 1.10 (0.57, 2.12) Treated in a hospital versus present facility: 0.80 (0.43, 1.48) Travel time to facility >1 h versus ≤1 h: **1.64 (0.96, 2.79)**			
Sub-Saharan Africa				
Clegg-Lamptey, 2009 [18] (Ghana)	**Reasons for delay from symptoms recognition to presentation at the study hospital where diagnosis was made (%)** Lack of BC awareness: 28.8% Fear of diagnosis or mastectomy: 34.8% Tried traditional/alternative treatments: 19.7% Tried spiritual cures: 19.7% Financial problems: 18.2% Lack of knowledge of BSE: 57.6% Other: 4.5% *Health services related:* Previous medical consultation: 39.4% Previous hospital consultations at a different hospital: 72.7%, with diagnosis made in only 52% of these			
Ezeome, 2010 [19] (Nigeria)	**Reasons for delay between symptoms recognition and visit to first HCP** Symptom(s) not serious/hoping they will resolve: 27.8% Lack of BC awareness: 23.3% Tried traditional/spiritual treatments: 12.6% Financial problems: 13.9% Painless: 12% Fear/refusal of mastectomy: 5.6% Family/social problems: 5.6% Though it was pregnancy/lactation effect: 3.2% Discouraged by friends/relatives: 3.2% Others: 15.7%	n/a	**Reasons for delay between symptoms recognition and start of BC treatment** *Patient-related* Lack of BC awareness: 25.3% Finance: 16.9% Thought it was harmless/will disappear: 15.4% Fear/refused surgery/mastectomy: 9.2% Painless/not disturbing her: 6.9% Delayed by family/social problems: 6.9% Traditional/spiritual treatments: 5.4% Discouraged by friends/relatives: 5.4% *Health care provider–related:* Delayed referrals or nonreferrals: 17.8% Wrong advice and false reassurance by health professionals: 11.5% Delayed histology report: 6.2% No histology after biopsy: 5.4% Industrial actions: 4.6%	n/a
Ibrahim, 2012 [20] (Nigeria)	**Delay from symptoms recognition to first medical consultation >3 mo** *Reasons given:* Lack of BC awareness: 34.1% Belief in spiritual healing: 32.3% Fear of mastectomy: 29.3% Belief in herbal treatment: 22% Belief in alternative therapy: 7.3% Lack of funds: 3% Reassurance by nonmedical health worker: 3% *Crude analysis:* Being single: OR = 2.05, 95% CI: 0.25, 16.8 Primary level of education: OR = 3.06, 95% CI: 0.96, 9.73 Negative history of benign breast disease: OR = 1.65, 95% CI: 0.76, 3.59	**Delay from symptoms recognition to first medical consultation >3 mo** “In the multivariate analysis, being premenopausal (OR = 1.86; 95% CI, 0.38, 9.4) was the additional factor associated with increased risk of late presentation” (sic)	n/a	n/a
Marcus, 2013 [21]† (South Africa)	**Delay from first symptoms to presentation >6 mo versus 3–6 mo: OR (95% CI)** *Socioeconomic:* Age (vs. 34–45 [sic]): 45–54: 0.15 55–64: 0.18 65–83: 0.77 Education (vs. none): Primary: 0.41 Secondary or higher: 0.18 Employed versus unemployed: 0.26 Married versus single/divorced/widowed: 0.31 *BC awareness:* Previous cancer diagnosis: 0	Delay from first symptoms to presentation >6 mo versus 3–6 mo: Adjusted* OR (95% CI) *Socioeconomic*: Age (vs. 34–45 [sic]): 45–54: 2.05 55–64: **2.55 (*P* < .05)** 65–83: 2.28 Education (vs. none): Primary: 0.27 Secondary or higher: 1.56 Employed versus unemployed: 0.63 Married versus single/divorced/widowed: 0.84 *BC awareness:* Previous cancer diagnosis: 22.13 (*P* < .01) *for all variables in the model	n/a	n/a
Otieno, 2010 [22]† (Kenya)	**Reasons for delays from first symptoms to presentation at diagnostic hospital, i.e., to diagnosis** Lack of BC awareness: 7.8% Painless symptom(s): 23.5% Fear of cancer: 19.9% Symptoms considered benign by health professionals: 24.1% Tried traditional treatments: 9.6% Others: 15.1%			
Pace, 2015 [23] (Rwanda)	**Reasons for delay between first symptoms to first visit to an HCP (%)** Did not think it was a problem at first: 76% Thought it would go away: 63% Visited traditional healer first: 21% Thought treatment was too expensive: 14% Too busy at home or job: 7% Fear of cancer: 6% Afraid of treatment and mastectomy: 5%	Reasons for delay between first symptoms to first visit to an HCP: OR* (95% CI) for delay >6 versus <6 mo *Socioeconomic:* Age (y) versus <40 y: 40–49: 2.26 (0.69, 7.43) 50–59: 1.22 (0.36, 4.11) >60: 2.30 (0.60, 8.74) Married versus unmarried: 1.11 (0.51, 2.48) No education/primary school versus secondary/university: **4.88 (1.72, 13.88)** *Reproductive:* Breastfeeding (yes vs. no): 2.09 (0.44, 9.87) *BC awareness:* BC family history (yes vs. no): 0.53 (0.14, 2.04) Ever done BSE (yes vs. no): 0.73 (0.31, 1.74) Ever heard of BC (yes vs. no): 1.86 (0.69, 5.00) *Type of symptoms and comorbidities:* Breast pain as initial symptom (yes vs. no): 0.57 (0.25, 1.30) HIV or other comorbidities (yes vs. no/unknown): 1.15 (0.43, 3.07) *Alternative treatments:* Saw traditional healer first: **4.26 (1.56, 11.60)** *Health services related:* Travel time to HCP (>2 vs. ≤2 h): 0.96 (0.36, 2.57) Regular CHW visits (yes vs. no): 1.51 (0.66, 3.46) *Mutually adjusted for all variables in the model	**Reasons for delay between first visit to an HCP and date of pathology report confirming BC (%)** Nonreferral from another health care center: 69% Did not know this cancer existed: 30% Did transfer from another health facility: 27% Too expensive to travel from home to hospital: 21% Told by a health care provider, there was no cure: 3% Hospital too far to travel to: 2%	**Reasons for delay between first visit to an HCP and date of pathology report confirming BC** OR* (95% CI) for delay ≥6 versus 6 mo *Socioeconomic:* Age (y) versus <40 y: 40–49: 0.57 (0.20, 1.68) 50–59: 0.85 (0.28, 2.62) >60: 0.64 (0.18, 2.24) Married versus unmarried: 1.11 (0.51, 2.41) No education/primary school versus secondary/university: 1.19 (0.48, 2.97) *Reproductive:* Breastfeeding (yes vs. no): 0.81 (0.15, 4.30) *BC awareness:* BC family history (yes vs. no): 0.60 (0.15, 2.34) Ever done BSE (yes vs. no): 1.15 (0.50, 2.65) Ever heard of BC (yes vs. no): 1.19 (0.45, 3.10) *Type of symptoms and comorbidities:* Breast pain as initial symptom (yes vs. no): 1.15 (0.52, 2.55) HIV or other comorbidities (yes vs. no/unknown): 0.84 (0.32, 2.17) *Health services related:* Travel time to an HCP (>2 vs. ≤2 h): 1.26 (0.46, 3.42) Regular CHW visits (yes vs. no): 1.14 (0.50, 2.58) No. of visits to other health care facilities before diagnosis (<5 vs. ≥5): **2.69 (1.24, 5.84)** Referred by (vs. health center): District hospital: 0.51 (0.09, 2.78) Private hospital: 0.36 (0.06, 2.09) Unknown: 0.49 (0.07, 3.45) *Mutually adjusted for all variables in the model
Price, 2012 [24] (Cameroon)	Financial problems: 16% Spent >$10 on one-way transportation: 42% Traveled >4 h to hospital: 46%		n/a	
Toure, 2013 [25] (Côte d’Ivoire)	**Reasons for delay between symptoms recognition and date of histological confirmation**			
	**Crude OR (95% CI) for delay > 6 mo** *Initial symptom (vs. nodule)* Inflammation: **23.6 (7.5, 74.0)** Ulcer: **18.1 (4.3, 76.9)** Nipple discharge: 1.9 (0.6, 6.2) Metastases: **13.9 (3.3, 59.3)** *Self-reported reason for delay (vs. having financial problems)* Traditional medicine: 0.5 (0.2, 1.2) Fear of cancer: 0.4 (0.1, 2.3) Misdiagnosis: 1.8 (0.2, 15.3) Inadequate medical care: 1.1 (0.2, 5.4) *Monthly income in euros (vs. none):* <91.46: 1.4 (0.5, 3.6) 91.46–182.8: 0.8 (0.3, 2.1) 182.9–274.4: 0.8 (0.3, 2.3) >274.4: 3.2 (0.4, 25.9)		**Mutually adjusted OR (95% CI) for delay >6 mo** (having financial problems taken as the reference category) *Self-reported reason for delay (vs. having financial problems)* Traditional medicine: 0.7 (0.7, 3.2) Fear of cancer: 1.2 (0.0, 12.3) Misdiagnosis: 3.0 (0.3, 5.7) Inadequate medical care: 0.6 (0.1, 17.4) *Monthly income in euros (vs. none):* <91.46: 0.3 (0.0, 1.7) 91.46–182.8: 4.4 (0.2, 91.2) 182.9–274.4: 12.7 (0.4, 376.6) >274.4: 47.8 (0.7, 3.103 [sic])	
Quantitative and qualitative studies				
Dye, 2010 [26] (Ethiopia)	**Reasons for delays between symptom recognition and presentation at diagnostic center (TA)** Lack of BC awareness *Health services:* High travel distance Too expensive >3 HCP visits: 73.2% *First HCP: % of patients (Me ± SE number of care nodes visited including study setting [TAH]):* Primary care: 53.7% (3.3 ± 1.8) Traditional healer: 16.4% (3.8 ± 0.26) Local/regional hospital: 16.4% (2.3 ± 0.19) Private hospital: 9% (2.8 ± 0.48) TAH: 4.5%			
Ly, 2002 [27] (Mali)	**Reasons for delays between symptom recognition and presentation at first HCP** Symptom(s) not serious: 82% Caused by witchcraft: 14%		n/a	
**Qualitative studies**				
Ekortarl, 2007 [28] (Cameroon)	**Reasons for delays between symptom recognition and presentation at first HCP** Ignorance and beliefs Fears Financial problems Inadequate diagnosis by general doctors			
Mbuka-Ongona, 2012 [29] (Botswana)	**Reasons for delays between symptom recognition and visit to diagnostic center** Lack of BC awareness Misinterpretation of signs Infrequent BSE Fear of diagnosis and death Influence of traditional healers *Health services:* Poor clinical practices of health workers Overemphasis on HIV infection Long travel distance to hospital			
Pruitt, 2015 [30] (Nigeria)	**Reasons for delays between symptom recognition and first visit to an HCP** Lack of BC awareness Symptom(s) not serious Tried traditional and spiritual treatments		**Reasons for delays between presentation and diagnosis and treatment** Inappropriate medical care given Delays in getting diagnostic confirmation or treatment Return to traditional care Denial Fear of surgery Strikes by hospital staff Treatment costs	

BC = breast cancer; BSE = breast self-examination; CBE = clinical breast examination; CHW = community health worker; CI = confidence interval; HCP = health care provider; HIV = human immunodeficiency virus; IQR = interquartile range; km = kilometers; LABC = locally advanced breast cancer; Md = mean; Me = mean; n/a = not reported in the original publication; OC = oral contraceptives; OR = odds ratio; Ra = range; SE = standard error; TAH = Tikur Anbessa Hospital; TCC = Tanca Cancer Center.

Bold values are statistically significant.

† Study recruited only patients with advanced breast cancer (see Table 1).

## Appendix

### Appendix B Quality assessment of the eligible articles

The quality of the articles included in the review was assessed by developing a standardized quality assessment form, which included parameters on three main domains: (1) Selection bias: study design (score 0 if unclear, 1 if retrospective case series, 2 if prospective study); study population (score 0 if unclear, 1 if opportunistic hospital-based study, 2 if consecutive hospital-based study, 3 if population-based study); restricted to late stage/advanced disease (score 0 if unclear, 1 if yes, 3 if no); participation rate (score 0 if unclear, 1 if <70%, 2 if ≥70%). (2) Information bias: source of the information (i) for patient-mediated factors (score 0 if unclear or n/a [not applicable], 1 if medical records, 2 if proxy [relative], 3 if patient); (ii) for health service–mediated factors (score 0 if unclear or n/a, 1 if proxy [relative], 2 if patient, 3 if medical records); timing of information collection (score 0 if unclear or n/a, 1 if after patient was aware of her breast cancer diagnosis, 2 if around the time of her diagnosis, 3 if before her diagnosis); potential of observer/interviewer bias (score 0 if unclear, 1 if likely, 2 if unlikely as information was validated against medical records or a previously validated questionnaire was used). (3) Analytical methods including dealing with potential confounders: definition of delays in presentation, diagnosis, and/or treatment (score 0 if not given, 1 if given but unclear or stage used as a proxy, 2 if clear); distinction between patient-related and health system–related factors (score 0 if not given, 1 if focus only on one of these, 2 if given but unclear, 3 if clear); statistical methods (score 0 if not properly described, 1 if only descriptive, 2 if analytical or in-depth); adjustment for potential confounders (score 0 if n/a, 1 if only crude estimates given, 2 if adjusted). The overall quality score of an article was expressed as the sum of its parameter-specific scores, which could range from 0 (lowest) to 30 (highest). The higher the score, the higher the methodological quality of the article and, hence, the lower the score, the more likely its findings might have been affected by biases.

## References

[1] Forouzanfar M.H., Foreman K.J., Delossantos A.M., Lozano R., Lopez A.D., Murray C.J. (2011). Breast and cervical cancer in 187 countries between 1980 and 2010: a systematic analysis. Lancet.

[2] Ferlay J., Shin H.R., Bray F., Forman D., Mathers C., Parkin D.M. (2010). Estimates of worldwide burden of cancer in 2008: GLOBOCAN 2008. Int J Cancer.

[3] Akarolo-Anthony S.N., Ogundiran T.O., Adebamowo C.A. (2010). Emerging breast cancer epidemic: evidence from Africa. Breast Cancer Res.

[4] Jedy-Agba E., McCormack V., Adebamowo C., Dos-Santos-Silva I. (2016). Stage at diagnosis of breast cancer in sub-Saharan Africa: a systematic review and meta-analysis. Lancet Glob Health.

[5] Brinton L.A., Figueroa J.D., Awuah B., Yarney J., Wiafe S., Wood S.N. (2014). Breast cancer in Sub-Saharan Africa: opportunities for prevention. Breast Cancer Res Treat.

[6] Richards M.A., Westcombe A.M., Love S.B., Littlejohns P., Ramirez A.J. (1999). Influence of delay on survival in patients with breast cancer: a systematic review. Lancet.

[7] Adebamowo C.A., Ajayi O.O. (2000). Breast cancer in Nigeria. West Afr J Med.

[8] Moher D., Liberati A., Tetzlaff J., Altman D.G. (2009). Preferred reporting items for systematic reviews and meta-analyses: the PRISMA statement. PLoS Med.

[9] Stapleton J.M., Mullan P.B., Dey S. (2011). Patient-mediated factors predicting early- and late-stage presentation of breast cancer in Egypt. Psychooncology.

[10] United Nations Statistics Division (2016). United Nations methods and classification: composition of macro geographical (continental) regions, geographical sub-regions, and selected economic and other groupings. https://unstats.un.org/unsd/methodology/m49/.

[11] Ahmed A.A. (2014). Clinicopathological profile of female Sudanese patients with locally advanced breast cancer. Breast Dis.

[12] Aloulou S., El Mahfoudi A., El Omrani A., Khouchani M. (2015). [Factors related to late diagnosis of breast cancer: experience of CHU Mohammed VI Marrakech]. Pan Afr Med J.

[13] Benbakhta B., Tazi M., Benjaafar N., Khattabi A., Maaroufi A. (2015). [Determinants of patient and health system delays for women with breast cancer in Morocco, 2013]. Rev Epidemiol Sante Publique.

[14] El-Shinawi M., Youssef A., Alsara M., Hablas A., Gaafar R., Seifeldin I.A. (2013). Assessing the level of breast cancer awareness among recently diagnosed patients in Ain Shams University Hospital. Breast.

[15] Ermiah E., Abdalla F., Buhmeida A., Larbesh E., Pyrhonen S., Collan Y. (2012). Diagnosis delay in Libyan female breast cancer. BMC Res Notes.

[16] Landolsi A., Gahbiche S., Chaafii R., Chabchoub I., Ben Fatma L., Hochlef M. (2010). [Reasons of diagnostic delay of breast cancer in Tunisian women (160 patients in the central region of Tunisia)]. Tunis Med.

[17] Mousa S.M., Seifeldin I.A., Hablas A., Elbana E.S., Soliman A.S. (2011). Patterns of seeking medical care among Egyptian breast cancer patients: relationship to late-stage presentation. Breast.

[18] Clegg-Lamptey J., Dakubo J., Attobra Y.N. (2009). Why do breast cancer patients report late or abscond during treatment in Ghana? A pilot study. Ghana Med J.

[19] Ezeome E.R. (2010). Delays in presentation and treatment of breast cancer in Enugu, Nigeria. Niger J Clin Pract.

[20] Ibrahim N.A., Oludara M.A. (2012). Socio-demographic factors and reasons associated with delay in breast cancer presentation: a study in Nigerian women. Breast.

[21] Marcus T.S., Lunda S., Fernandez L. (2013). Delayed breast cancer presentation: hospital data should inform proactive primary care. Afr J Prim Health Care Fam Med.

[22] Otieno E.S., Micheni J.N., Kimende S.K., Mutai K.K. (2010). Delayed presentation of breast cancer patients. East Afr Med J.

[23] Pace L.E., Mpunga T., Hategekimana V., Dusengimana J.M., Habineza H., Bigirimana J.B. (2015). Delays in breast cancer presentation and diagnosis at two rural cancer referral centers in Rwanda. Oncologist.

[24] Price A.J., Ndom P., Atenguena E., Mambou Nouemssi J.P., Ryder R.W. (2012). Cancer care challenges in developing countries. Cancer.

[25] Toure M., Nguessan E., Bambara A.T., Kouassi Y.K., Dia J.M., Adoubi I. (2013). [Factors linked to late diagnosis in breast cancer in Sub-Saharan Africa: case of Cote d'Ivoire]. Gynecol Obstet Fertil.

[26] Dye T.D., Bogale S., Hobden C., Tilahun Y., Hechter V., Deressa T. (2010). Complex care systems in developing countries: breast cancer patient navigation in Ethiopia. Cancer.

[27] Ly M., Diop S., Sacko M., Baby M., Diop C.T., Diallo D.A. (2002). [Breast cancer: factors influencing the therapeutic itinerary of patients in a medical oncology unit in Bamako (Mali)]. Bull Cancer.

[28] Ekortarl A., Ndom P., Sacks A. (2007). A study of patients who appear with far advanced cancer at Yaounde General Hospital, Cameroon, Africa. Psychooncology.

[29] Mbuka-Ongona D., Tumbo J.M. (2013). Knowledge about breast cancer and reasons for late presentation by cancer patients seen at Princess Marina Hospital, Gaborone, Botswana. Afr J Prim Health Care Fam Med.

[30] Pruitt L., Mumuni T., Raikhel E., Ademola A., Ogundiran T., Adenipekun A. (2015). Social barriers to diagnosis and treatment of breast cancer in patients presenting at a teaching hospital in Ibadan, Nigeria. Glob Public Health.

[31] Barros A.F., Uemura G., de Macedo J.L. (2013). [Interval for access to treatment for breast cancer in the Federal District, Brazil]. Rev Bras Ginecol Obstet.

[32] Norsa'adah B., Rampal K.G., Rahmah M.A., Naing N.N., Biswal B.M. (2011). Diagnosis delay of breast cancer and its associated factors in Malaysian women. BMC Cancer.

[33] Molinie F., Leux C., Delafosse P., Ayrault-Piault S., Arveux P., Woronoff A.S. (2013). Waiting time disparities in breast cancer diagnosis and treatment: a population-based study in France. Breast.

[34] Richardson L.C., Royalty J., Howe W., Helsel W., Kammerer W., Benard V.B. (2010). Timeliness of breast cancer diagnosis and initiation of treatment in the National Breast and Cervical Cancer Early Detection Program, 1996-2005. Am J Public Health.

[35] Allgar V.L., Neal R.D. (2005). Delays in the diagnosis of six cancers: analysis of data from the National Survey of NHS Patients. Cancer Br J Cancer.

[36] Arndt V., Sturmer T., Stegmaier C., Ziegler H., Dhom G., Brenner H. (2002). Patient delay and stage of diagnosis among breast cancer patients in Germany—a population based study. Br J Cancer.

[37] Arndt V., Sturmer T., Stegmaier C., Ziegler H., Becker A., Brenner H. (2003). Provider delay among patients with breast cancer in Germany: a population-based study. J Clin Oncol.

[38] Pineros M., Sanchez R., Perry F., Garcia O.A., Ocampo R., Cendales R. (2011). [Delay for diagnosis and treatment of breast cancer in Bogota, Colombia]. Salud Publica Mex.

[39] Unger-Saldana K., Miranda A., Zarco-Espinosa G., Mainero-Ratchelous F., Bargallo-Rocha E., Miguel Lazaro-Leon J. (2015). Health system delay and its effect on clinical stage of breast cancer: multicenter study. Cancer.

[40] Opoku S.Y., Benwell M., Yarney J. (2012). Knowledge, attitudes, beliefs, behaviour and breast cancer screening practices in Ghana, West Africa. Pan Afr Med J.

[41] Adisa A.O., Gukas I.D., Lawal O.O., Adesunkanmi A.R. (2010). Breast cancer in Nigeria: is non-adherence to chemotherapy schedules a major factor in the reported poor treatment outcome?. Breast J.

[42] Galukande M., Wabinga H., Mirembe F. (2015). Breast cancer survival experiences at a tertiary hospital in sub-Saharan Africa: a cohort study. World J Surg Oncol.

[43] Kantelhardt E.J., Zerche P., Mathewos A., Trocchi P., Addissie A., Aynalem A. (2014). Breast cancer survival in Ethiopia: a cohort study of 1,070 women. Int J Cancer.

[44] Yip C.H., Smith R.A., Anderson B.O., Miller A.B., Thomas D.B., Ang E.S. (2008). Guideline implementation for breast healthcare in low- and middle-income countries: early detection resource allocation. Cancer.

[45] Harirchi I., Kolahdoozan S., Karbakhsh M., Chegini N., Mohseni S.M., Montazeri A. (2011). Twenty years of breast cancer in Iran: downstaging without a formal screening program. Ann Oncol.

